# Comparative efficacy and safety of medical and surgical management for missed miscarriage: a systematic review and meta-analysis

**DOI:** 10.3389/fmed.2026.1801007

**Published:** 2026-04-15

**Authors:** Kaiyi Wei, Shijin Huang, Lingjie Deng, Yi Li, Yibao Huang, Lihong Pang

**Affiliations:** 1 Department of Prenatal Diagnosis, The First Affiliated Hospital of Guangxi Medical University, Nanning, China; 2 Department of Gynecology, The Second Affiliated Hospital of Guangxi Medical University, Nanning, Guangxi, China; 3 The First Affiliated Hospital, Center for a Combination of Obstetrics and Gynecology & Reproductive Medicine, Hengyang Medical School, University of South China, Hengyang, China; 4 Guangxi Key Laboratory of Thalassemia Research, Nanning, Guangxi, China; 5 National Health Commission Key Laboratory of Thalassemia Medicine (Guangxi Medical University), Nanning, Guangxi, China; 6 Guangxi Key Laboratory of Early Prevention and Treatment for Regional High Frequency Tumor, Nanning, Guangxi, China

**Keywords:** dilation and curettage, mifepristone, misoprostol, missed miscarriage, suction curettage, surgical management

## Abstract

**Background:**

Comparative evidence regarding the efficacy and safety of medical versus surgical management for missed miscarriage has not been consistently evaluated.

**Methods:**

The meta-analysis was in line with the PRISMA 2020 and MOOSE guidelines. The Web of Science, PubMed, Embase, and ScienceDirect databases were searched for eligible studies. The outcomes were the success rate, bleeding duration, infection rate and complication rate. The pooled results were synthesized via random-effect model. Influential publication was determined by performing sensitivity analysis. In addition, the potential sources of heterogeneity were examined by using subgroup analyses. Publication bias was assessed using the funnel plot, Begg’s and Egger’s tests.

**Results:**

The seven included studies (four RCTs and three cohort studies) were conducted between 1994 and 2025, with a total of 1,637 patients with missed miscarriage. We found that surgical management demonstrates superior clinical efficacy and safety compared to medical management, with higher success rate [risk difference (RD) = 0.26, 95% confidence interval (95% CI): 0.14 to 0.39, *P* < 0.001], shorter bleeding duration [weighted mean difference (WMD) = −2.72, 95% CI: −4.53 to −0.92, *P* < 0.001] and fewer complications (RD = −0.29, 95% CI: −0.43 to −0.15, *P* < 0.001). No significant difference in infection rate (RD = −0.02, 95% CI = −0.07 to 0.03, *P* = 0.404). Subgroup analysis showed that patients’ mean gestational week was the potential source of heterogeneity. No influential publications and significant publication bias were detected across studies.

**Conclusion:**

Surgical management demonstrates superior clinical efficacy and safety compared to medical management, especially in pregnancies with earlier gestational age. A truly patient-centered approach must balance individual preferences, gestational age, follow-up access, and awareness of clinical risks.

## 1 Introduction

Missed miscarriage, also known as missed abortion, is a form of early pregnancy loss in which embryonic or fetal demise occurs without the immediate expulsion of gestational tissue from the uterine cavity (1). Notably, missed miscarriage often presents without the classic symptoms of miscarriage, such as vaginal bleeding or uterine cramping, frequently leading to delayed diagnosis and potential complications.

The etiology of missed miscarriage is multifactorial, with chromosomal abnormalities accounting for 56%–60% of cases. Additional contributing factors include maternal immune disorders, infections, structural uterine anomalies, and endocrine imbalances (2). For women diagnosed with missed miscarriage who are hemodynamically stable and without signs of infection, three primary management strategies are available: expectant, medical, and surgical management (3).

Expectant management, recommended as first-line care in the United Kingdom with a 7–14 days observation window, relies on spontaneous expulsion of retained products of conception (4). However, its success rate varies widely (39%–75%) and is associated with risks of prolonged bleeding, unpredictable timing, and unplanned surgical intervention (5). Consequently, many patients and clinicians favor more definitive options.

Medical management, particularly using a combination of mifepristone and misoprostol, is widely endorsed in international guidelines and preferred by many women due to its non-invasive nature (3). Reported efficacy rates for this regimen range from 52% to 95%, which stems largely from heterogeneity in study protocols, dosing regimens, routes of administration, and outcome definitions (6). For example, vaginal administration of misoprostol appears to be more effective and associated with fewer side effects than oral administration (7). Despite its overall safety, medical abortion can be complicated by significant vaginal bleeding, and 15.2% of women required emergency surgical intervention for hemorrhage control (8). Moreover, 15%–40% of patients experience incomplete or failed abortion, necessitating either a second dose or eventual surgical evacuation (9).

In contrast, surgical management, typically performed via vacuum aspiration or uterine curettage, offers a high success rate exceeding 98% (10) and provides immediate resolution of the miscarriage, thereby minimizing the need for prolonged monitoring and reducing uncertainty (11). The World Health Organization (WHO) recognizes both surgical evacuation and medical abortion as safe and effective methods for first-trimester pregnancy termination (12). Nevertheless, robust comparative evidence regarding their effectiveness and safety have not been consistently evaluated across high-quality studies.

Given these uncertainties, this meta-analysis aims to systematically compare surgical and medical management with respect to four critical outcomes: treatment success rate, postoperative bleeding duration, infection rate, and overall complication rate, thereby informing evidence-based care.

## 2 Methods

The study was in line with Preferred Reporting Items for Systematic Reviews and Meta-Analysis (PRISMA) and MOOSE Guidelines (13, 14). The protocol for this systematic review and meta-analysis was registered on PROSPERO website (ID: CRD420261281331).

### 2.1 Eligibility criteria

Studies on comparing the clinical efficacy and safety of medical management versus surgical management for missed miscarriage will be included. Missed miscarriage was defined according to standard ultrasound criteria, including a gestational sac ≥ 25 mm without an embryo, lack of growth over at least 1 week, or a crown-rump length ≥ 7 mm without cardiac activity. Medical management was broadly defined to encompass various pharmacological regimens, including the combination of mifepristone and misoprostol, misoprostol alone, or other abortifacient agents. Surgical management included procedures for uterine evacuation such as suction curettage, dilation and curettage (D&C), and related surgical techniques. Studies that meet one of the following criteria were excluded: (1) laboratory research; (2) non-English articles; and (3) no full text available. Publication date, study design and sample size were not considered as the basis for exclusion.

### 2.2 Search strategy

According to the PICO strategy for the review question, the keywords included adult female with missed miscarriage (P), medical management versus surgical management (I), and clinical efficacy and safety (O). The Web of Science, PubMed, ScienceDirect, and Embase databases were screened from their inception up to January 7th, 2026. References in the reviews and meta-analysis about this topic were also searched. The search query was: {(missed miscarriage or missed abortion or delayed miscarriage) and (mifepristone or misoprostol or medical abortion or medical management or pharmacological treatment or drug therapy or medical treatment) and [(dilation and curettage) or D&C or surgical evacuation or uterine curettage or vacuum aspiration or surgical management or surgical treatment]}. Specific retrieval strategies for each database were shown in Supplementary Table 1.

### 2.3 Study selection

After searching out the retrieved studies separately by databases, duplicate items were identified and then removed using EndNote software. Then, the titles and abstracts were reviewed for relevance to the principal purpose of this study. Finally, the full text was reviewed according to inclusion and exclusion criteria. Two reviewers independently screened records, with disagreements resolved through discussion or adjudication by a third reviewer.

### 2.4 Quality assessment

Two reviewers independently evaluated the risk of bias of the 4 RCTs using the version 2 of the Cochrane risk-of-bias (RoB 2) tools (15) and quality of three observational studies was evaluated using the Newcastle-Ottawa Scale (NOS) checklist (16). The NOS checklist comprises seven items, each of which gets one point except the comparison item which can get a maximum of two points. Studies scoring below five were considered at high risk of bias.

### 2.5 Data extraction and synthesis

Two independent reviewers performed the data extraction, with a third reviewer double-checking the extracted information. Data were extracted using a standardized form covering author, year, country, simple size, mean age, gestational week, medication group, surgical group, follow-up, and clinical outcome. The clinical outcome in this study included success rate, bleeding duration, infection rate and complication rate. Effect sizes were expressed as weighted mean difference (WMD) for continuous outcomes and risk difference (RD) for dichotomous outcomes, each with 95% confidence interval (95% CI). Heterogeneity was quantified by I^2^ statistic and Cochran’s Q test. The values of 25%, 50%, and 75% were correspond to cut-off points for low, moderate, and high heterogeneity, respectively. Random-effect model was chosen to compute in moderate or high heterogeneity; otherwise, a fixed-effect model was adopted. Due to the high heterogeneity, the sensitivity analysis was adopted to detect and then eliminate the influential publications. In addition, potential source of heterogeneity was identified by subgroup analysis. Publication bias was assessed by using the funnel plot, Begg’s and Egger’s tests.

### 2.6 Statistical analysis

The pooled results were executed with STATA-16 software by employing “metan,” “metaninf,” “metafunnel,” and “metabias” packages. All tests were two-tailed, and a *P*-value < 0.05 was considered to be statistically significant.

## 3 Results

This study presents a comprehensive meta-analysis comparing the clinical efficacy and safety of medical versus surgical management in patients with missed miscarriage, while also exploring potential factors associated with treatment outcomes.

### 3.1 Study selection

**FIGURE 1:**
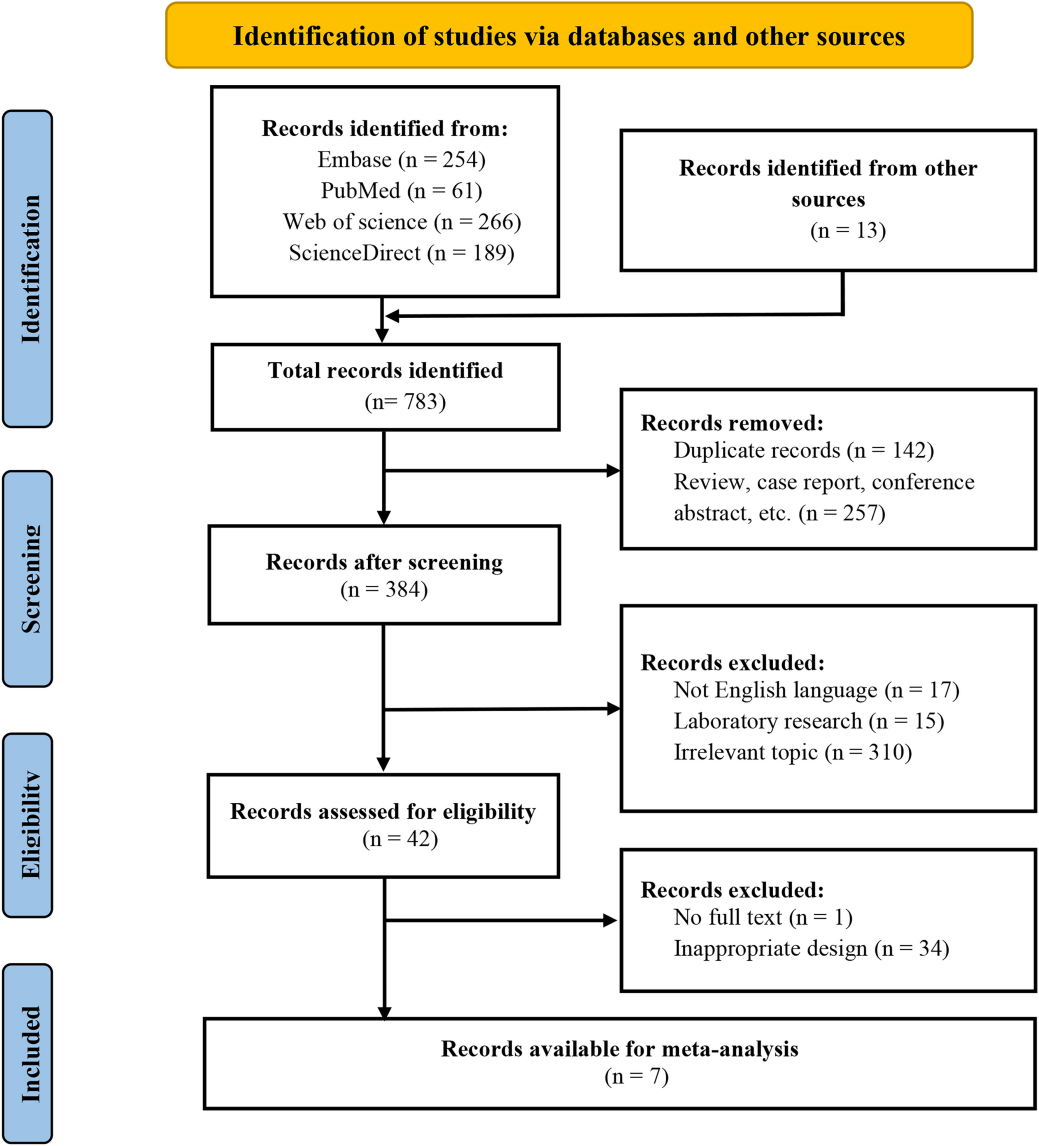
Preferred Reporting Items for Systematic Reviews and Meta-Analysis (PRISMA) flowchart of seven selected studies.

The selection process flow chart is presented in Figure 1. Initially, 770 potential citations were identified from the databases and 13 additional studies via hand-searching reference lists. After exclusion of irrelevant articles based on the titles and abstracts, 42 articles were assessed for eligibility. Following full-text screening, seven articles were included in the final analysis, which including seven studies for success rate (17–23), three studies for bleeding duration (17, 20, 21), three studies for infection rate (18, 19, 21), and three studies for complication rate (18, 20, 23).

### 3.2 Study characteristics and quality assessment

The seven included studies (four RCTs and three cohort studies) were conducted between 1994 and 2025, with a total of 1,637 patients. The sample size varied between 87 and 702 patients. The patients’ mean age ranged from 28.9 to 32.7 years, and gestational age at treatment ranged from 7.1 to 10.4 weeks. In the medical group, interventions primarily consisted of combination therapy with mifepristone and misoprostol (five studies); three studies used misoprostol or prostaglandins alone. In the surgical group, interventions included suction curettage (four studies) or D&C (three studies). Other detailed information about study characteristics was presented in Table 1.

**TABLE 1:** Summarized characteristics of seven included studies.

References	Country	Design	Simple size	Mean age (years)	Gestational week	Medical group	Surgical group	Follow-up (days)	Outcome
Bai et al. (17)	China	Cohort	702	32.6	9.8	Mf + Ms	Suction curettage	30	➀ ➁
Barghazan et al. (18)	Iran	RCT	168	32.7	9.7	Misoprostol	D & C	14	➀ ➂ ➃
Chia et al. (19)	UK	RCT	200	28.9	9.8	Mf + Ms	Suction curettage	7	➀ ➂
Egarter et al. (20)	Austria	RCT	87	30.6	10.1	Prostaglandin E	D & C	30	➀ ➁ ➃
Gronlund et al. (21)	Denmark	RCT	176	32.0	10.4	Misoprostol, Mf + Ms	Suction curettage	14	➀ ➁ ➂
Lei et al. (22)	China	Cohort	124	31.1	9.6	Mf + Ms	Suction curettage	14	➁
Torres-Miranda (23)	Spain	Cohort	180	32.5	7.1	Mf + Ms	D & C	5	➀ ➃

➀ Success rate; ➁ Bleeding duration; ➂ Infection rate; ➃ Complication rate; D & C, dilation and curettage; Mf + Ms, mifepristone and misoprostol; RCT, randomized controlled trial.

The quality of the four included RCTs (18–21) was evaluated via RoB 2 tool. Due to the treatment nature, it was difficult to blind study participants and to eliminate deviations from the intended intervention. Thus, all the included RCTs were deemed to have some concern risk of bias (Supplementary Figure 1). The quality of the three cohort studies was assessed by using the NOS checklist. Based on the quality score, the three cohort studies scored no less than five, and no study was excluded (Supplementary Table 2).

### 3.3 Surgical management offers higher success rate, shorter bleeding duration, and fewer complications

#### 3.3.1 Surgical management has higher success rate for missed miscarriage

**FIGURE 2:**
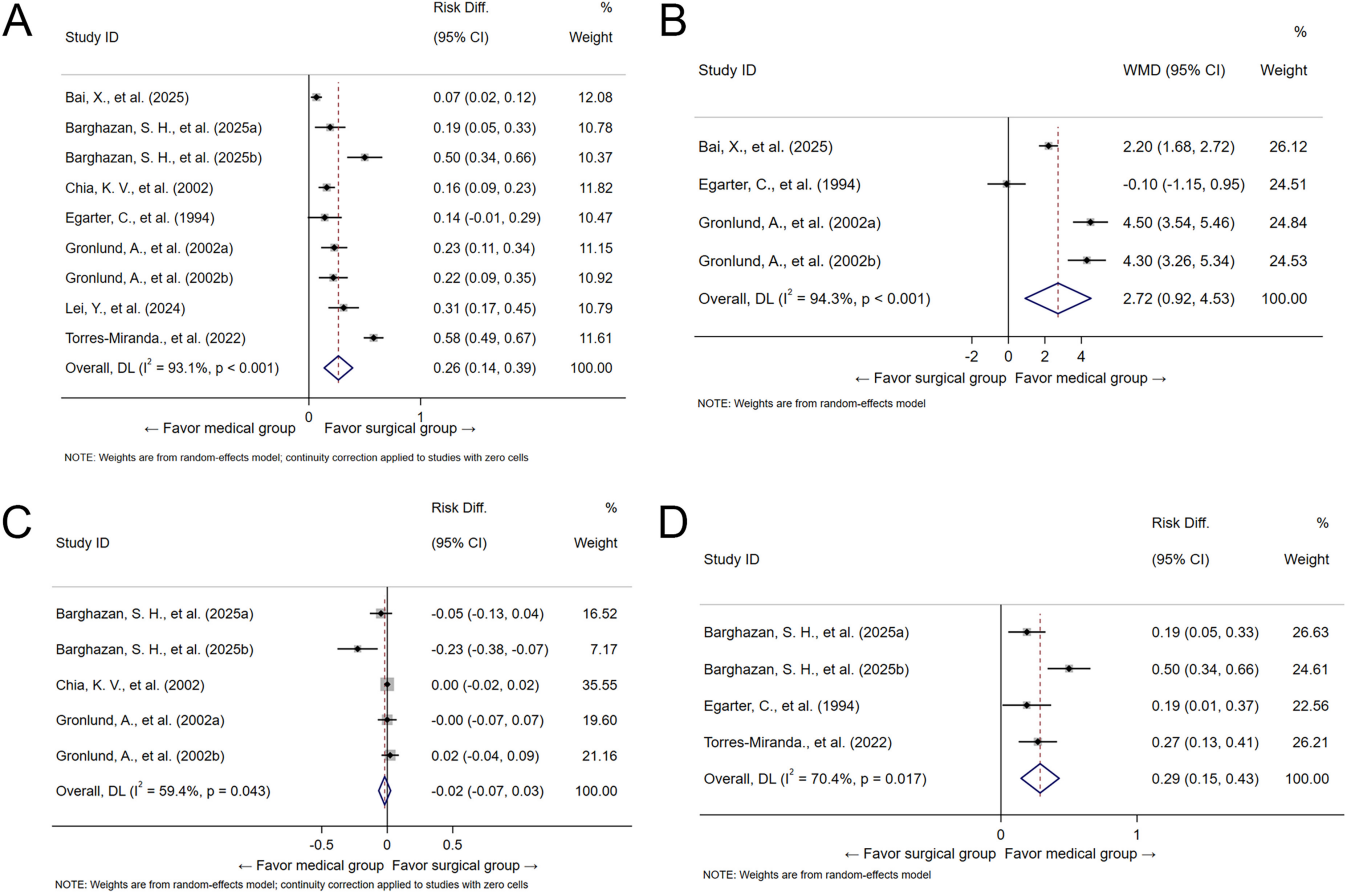
Forest plot of pooled risk difference (RD) for success rate **(A)**, weighted mean difference (WMD) for bleeding duration **(B)**, RD for infection rate **(C)**, and complication rate **(D)** comparing surgical versus medical management.

A total of 7 studies (17–23) were included in the analysis of success rate via random effect model, and the success rate from the surgical group were higher than the medical group (RD = 0.26, 95% CI = 0.14 to 0.39, *P* < 0.001). Heterogeneity among these studies was high (I^2^ = 93.1%, *P* < 0.001) (Figure 2A). However, no influential publications were detected among the pooled results (Supplementary Figure 2A). The results of the subgroup analysis showed that patients’ mean gestational week was the potential source of heterogeneity, whereas the study design, type of surgical or medical intervention was not (Table 2). Furthermore, no significant publication bias (Supplementary Figure 3A; Begg’s test: *z* = 0.73, *p*-value = 0.466; Egger’s test: *t* = 0.73, *p*-value = 0.467) were detected.

**TABLE 2:** Subgroup analyses for success rate, bleeding duration, infection rate, and complication rate comparing surgical versus medical management.

Outcome	Potential factors	Effect (95% CI)	I^2^	Interaction *p*-value
Success rate	Design = cohort	0.32 (0.03, 0.67)	98.0%	0.639
	Design = RCT	0.23 (0.14, 0.32)	68.3%	
	Mean gestational week ≤ 9.7	0.40 (0.21, 0.59)	88.6%	**0.016**
	Mean gestational week > 9.7	0.15 (0.08, 0.22)	63.6%	
	Surgical group = suction curettage	0.18 (0.10, 0.27)	77.7%	0.167
	Surgical group = dilation and curettage	0.36 (0.13, 0.58)	91.9%	
	Medical group = Combination	0.17 (0.08, 0.27)	80.3%	0.153
	Medical group = monotherapy	0.33 (0.14, 0.52)	91.1%	
Bleeding duration	Design = cohort	2.20 (1.68, 2.72)	0.0%	0.640
	Design = RCT	2.90 (−0.00, 5.81)	95.9%	
	Mean gestational week ≤ 10.1	1.10 (−1.16, 3.35)	93.3%	**0.006**
	Mean gestational week > 10.1	4.41 (3.70, 5.11)	0.0%	
	Surgical group = suction curettage	3.62 (1.96, 5.29)	91.8%	**<0.001**
	Surgical group = dilation and curettage	−0.10 (−1.15, 0.95)	0.0%	
	Medical group = combination	3.20 (1.14, 5.26)	92.0%	0.694
	Medical group = monotherapy	2.20 (−2.30, 6.71)	97.5%	
Infection rate	Mean gestational week ≤ 9.7	−0.12 (−0.30, 0.05)	74.9%	0.155
	Mean gestational week > 9.7	0.00 (−0.02, 0.02)	0.0%	
	Surgical group = suction curettage	0.00 (−0.02, 0.02)	0.0%	0.155
	Surgical group = dilation and curettage	−0.12 (−0.30, 0.05)	74.9%	
	Medical group = combination	0.00 (−0.02, 0.02)	0.0%	0.176
	Medical group = monotherapy	0.07 (−0.17, 0.03)	70.8%	
Complication rate	Design = cohort	0.27 (0.13, 0.41)	0.0%	0.858
	Design = RCT	0.29 (0.09, 0.50)	80.2%	
	Mean gestational week ≤ 9.7	0.32 (0.14, 0.49)	77.3%	0.320
	Mean gestational week > 9.7	0.19 (0.01, 0.37)	0.0%	
	Follow-up = 14 days	0.34 (0.04, 0.65)	88.2%	0.532
	Surgical group = other	0.24 (0.13, 0.35)	0.0%	
	Medical group = misoprostol	0.32 (0.14, 0.49)	77.3%	0.320
	Medical group = prostaglandin	0.19 (0.01, 0.37)	0.0%	

RCT, randomized controlled trial. Bold values represent the statistically significant interaction *p*-values (*p* < 0.05), which indicate the potential source of heterogeneity for the corresponding outcome indicators in the subgroup analysis.

#### 3.3.2 Surgical management has shorter bleeding duration after missed miscarriage

The three studies (17, 20, 21) evaluated the association between intervention type and bleeding duration via a random effect model. The bleeding duration from the medical group were longer compared to the surgical group (WMD = 2.72, 95% CI: 0.92 to 4.53, *P* < 0.001). In addition, there was high heterogeneity among the included studies (I^2^ = 94.3%, *P* < 0.001) (Figure 2B). There no influential publications among the pooled results (Supplementary Figure 2B). The subgroup analysis patients’ mean gestational week and type of surgical intervention were the potential source of heterogeneity, but the study design and type of medical intervention was not (Table 2). The funnel plot (Supplementary Figure 3B), Begg’s (*z* = −1.02, *p*-value = 1.000) and Egger’s (*t* = 0.11, *p*-value = 0.915) tests both indicated the low probability of publication bias.

#### 3.3.3 Surgical management carries a similar risk of infection to medical abortion

The association between intervention type and infection risk was evaluated in three studies (18, 19, 21) using a random-effects model. The pooled results showed that no significant difference in infection rate between surgical and medical group (RD = −0.02, 95% CI = −0.07 to 0.03, *P* = 0.404). There was significant heterogeneity across these studies (I^2^ = 59.4%, *P* = 0.043) (Figure 2C). No influential publications were detected (Supplementary Figure 2C). No significant sources of heterogeneity were identified in the subgroup analyses (Table 2). In addition, no potential publication bias (Supplementary Figure 3C, Begg’s test: *z* = −2.69, *p*-value = 1.000; Egger’s test: *t* = −1.93, *p*-value = 0.054) were discerned across studies.

#### 3.3.4 Surgical management has fewer complications after missed miscarriage

A total of three studies (18, 20, 23) evaluating the impact of intervention type on complication rate were included. As shown in Figure 2D, the medical group was found to be associated with significantly higher complication rate (RD = 0.29, 95% CI = 0.15 to 0.43, *P* < 0.001). Besides, significant heterogeneity was detected for these included studies (I^2^ = 70.4%, *P* = 0.017). However, no influential publications were detected (Supplementary Figure 2D). The subgroup analysis showed no possible sources of heterogeneity (Table 2). In addition, no significant publication bias (Supplementary Figure 3D, Begg’s test: *z* = 0.34, *p*-value = 0.734; Egger’s test: *t* = 0.07, *p*-value = 0.944) were discerned across studies.

## 4 Discussion

Missed miscarriage represents a common yet emotionally challenging form of early pregnancy loss, for which both surgical and medical management are widely used. Despite their routine application, high-quality comparative evidence on their relative efficacy and safety has remained inconsistent.

The present meta-analysis provides compelling evidence that surgical management is significantly more effective and safer than medical management among women with missed miscarriage. It achieves a substantially higher success rate (26%), reduces bleeding duration by nearly 3 days, and is associated with fewer overall complications. These findings support surgery as the preferred option for patients prioritizing treatment certainty, rapid resolution, and minimization of adverse events, particularly for pregnancies with earlier gestational age. Notably, infection rates did not differ significantly between approaches, alleviating a common concern about invasive intervention. These findings align with the mechanistic differences between the two approaches and offer important insights for clinical decision-making. However, while surgical management offers clear efficacy advantages, the choice of treatment must remain individualized.

Surgical evacuation achieves rapid and complete removal of retained products of conception, thereby minimizing prolonged bleeding and reducing the risk of secondary interventions. Our pooled results confirm a consistently superior efficacy of surgical intervention, particularly in pregnancies with earlier gestational age, where uterine size and tissue volume may compromise the effectiveness of pharmacological agents (24). Notably, international guidelines recommend different management approaches based on gestational age: medical abortion is often preferred before 7 weeks, whereas surgical evacuation is recommended for pregnancies between 7 and 14 weeks (25). This nuanced approach acknowledges that treatment efficacy is highly dependent on gestational age, which emerged in our analysis as a key source of heterogeneity. Indeed, studies enrolling participants with amenorrhea exceeding 11 weeks reported higher hemorrhage risks (8) and greater likelihood of surgical conversion, further supporting gestational age as a critical effect modifier. Moreover, the surgical group had a shorter duration of bleeding compared to medical group, consistent with findings from other studies (17, 20, 21). The shorter bleeding duration observed with surgery not only improves patient comfort but may also reduce psychological distress, a critical consideration given the high prevalence of anxiety and depression among women experiencing missed miscarriage (26).

However, the advantages of surgical management must be weighed against its potential iatrogenic harms. The re-evacuation rate following surgical management of incomplete miscarriage ranges from 1.1% to 2.1%, and the overall complication rate associated with the surgical approach is approximately 6.6% (5). Notably, instrumentation of the uterine cavity can cause mechanical trauma to both the functional and basal layers of the endometrium (27), disrupting normal regenerative processes and increasing the risk of intrauterine adhesions (IUA) (28). IUA, a well-documented sequela of post-miscarriage curettage (29), is associated with long-term reproductive morbidity, including infertility, amenorrhea, hypomenorrhea, and recurrent pregnancy loss (30). Despite the clear short-term benefits of surgery demonstrated in this meta-analysis, the long-term reproductive implications warrant caution. While estradiol supplementation post-curettage may mitigate fibrosis and support endometrial regeneration (31), it does not eliminate the fundamental risk inherent to uterine instrumentation. Therefore, many women prefer a less invasive approach to managing missed abortion and wish to remain awake during treatment, even if it means experiencing more pain and longer bleeding (21).

Although medical management entails longer bleeding and a higher rate of incomplete abortion requiring rescue surgery (17), it avoids direct endometrial trauma and remains a fertility-sparing option for many women. It preserves endometrial integrity by avoiding mechanical disruption, thereby offering a protective effect against IUA formation (17). In this situation, medical treatment was recommended over evacuation curettage due to its acceptable success rates, mild side effects, manageable complications, and higher patient satisfaction (10). The waiting time for the operating theater may sometimes extend to several days, whereas medical treatment can be initiated immediately after diagnosis. Additionally, medical regimens are associated with lower societal costs and better quality-of-life outcomes compared to surgical intervention (18), with an incremental cost-effectiveness ratio of £6,969 per additional successfully managed miscarriage (32), making them especially appealing in resource-limited settings or for patients prioritizing future fertility.

In summary, while current evidence suggests that surgical management offers superior immediate efficacy and fewer acute complications, the choice between surgical and medical approaches should not be based on efficacy alone. A truly patient-centered strategy must integrate individual preferences, gestational age, reproductive goals, access to follow-up care, and awareness of both short- and long-term risks.

## 5 Limitation

Although above important findings in this study, there are also some limitations. First, the degree of efficacy is closely related to the intervention protocols. But various management schemes cannot be maintained the same in different studies. This is one of the reasons why the heterogeneity was large in these studies. Second, although the study design are RCTs or cohort studies, the total number of included studies and specific subgroup was relatively small, especially for safety data, which may be problematic in terms of power and responsiveness. Third, although the inclusion of older studies provides valuable evidence, their generalizability to current clinical practice should be interpreted with caution given advances in treatment protocols over time. Therefore, future large-scale, pragmatic randomized trials with standardized protocols and long-term fertility outcomes are urgently needed to resolve existing uncertainties.

## 6 Conclusion

Surgical management is more effective and safer than medical management for missed miscarriage, with higher success rates, shorter bleeding duration, and fewer complications, especially at earlier gestational ages. However, the choice between surgical and medical approaches should not be based on efficacy alone. The patient-centered approach must balance clinical evidence with individual preferences, reproductive goals, access to follow-up, and awareness of risks to guide shared decision-making.
