# Supplementary figures and images for: Comparative efficacy and safety of medical and surgical management for missed miscarriage: a systematic review and meta-analysis
Source: Front Med (Lausanne). 2026 Apr 15;13:1801007. doi: 10.3389/fmed.2026.1801007 (PMC13125087; doi:10.3389/fmed.2026.1801007)

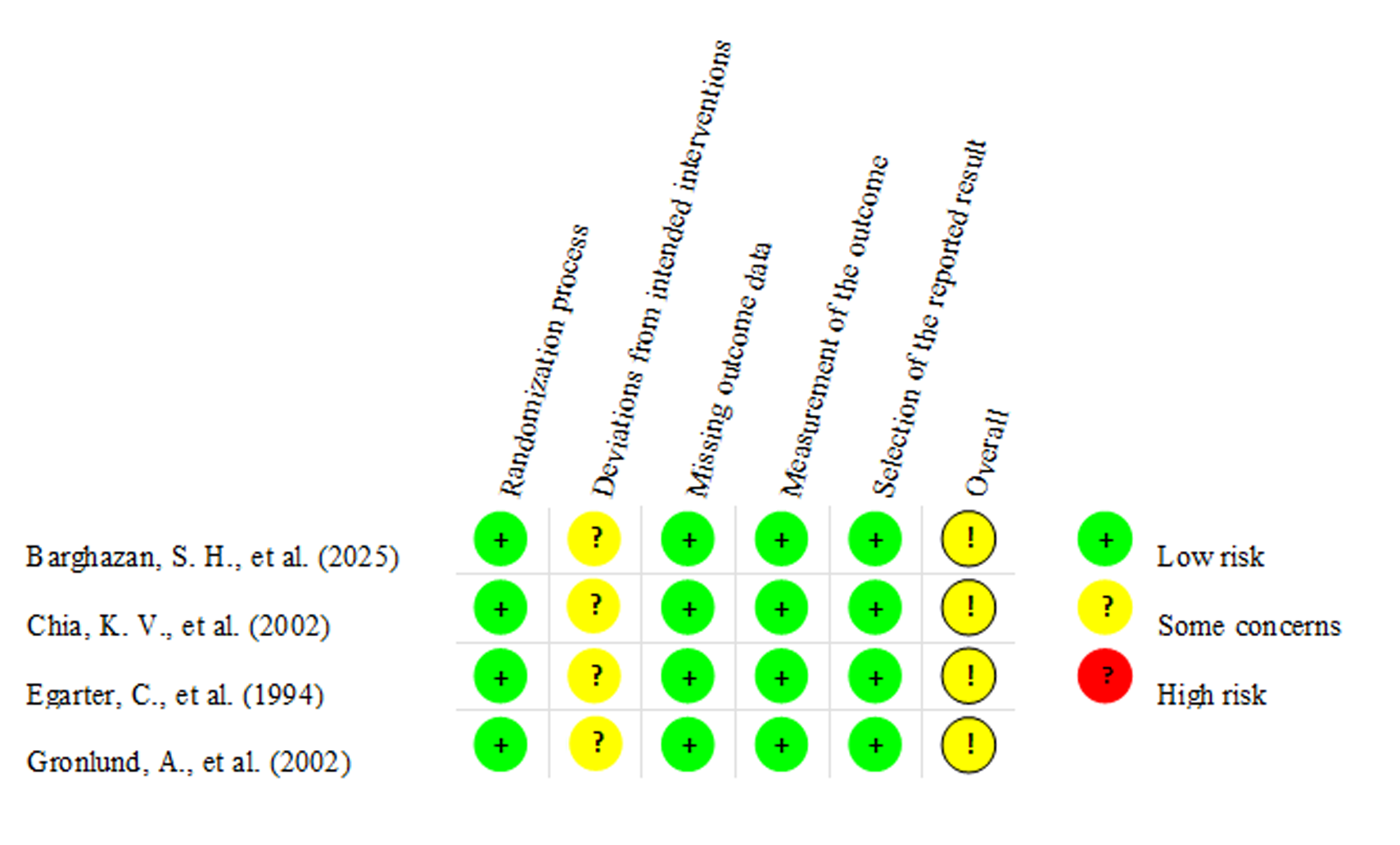

Supplement: Supplementary file 1 [file Image_1.TIF]

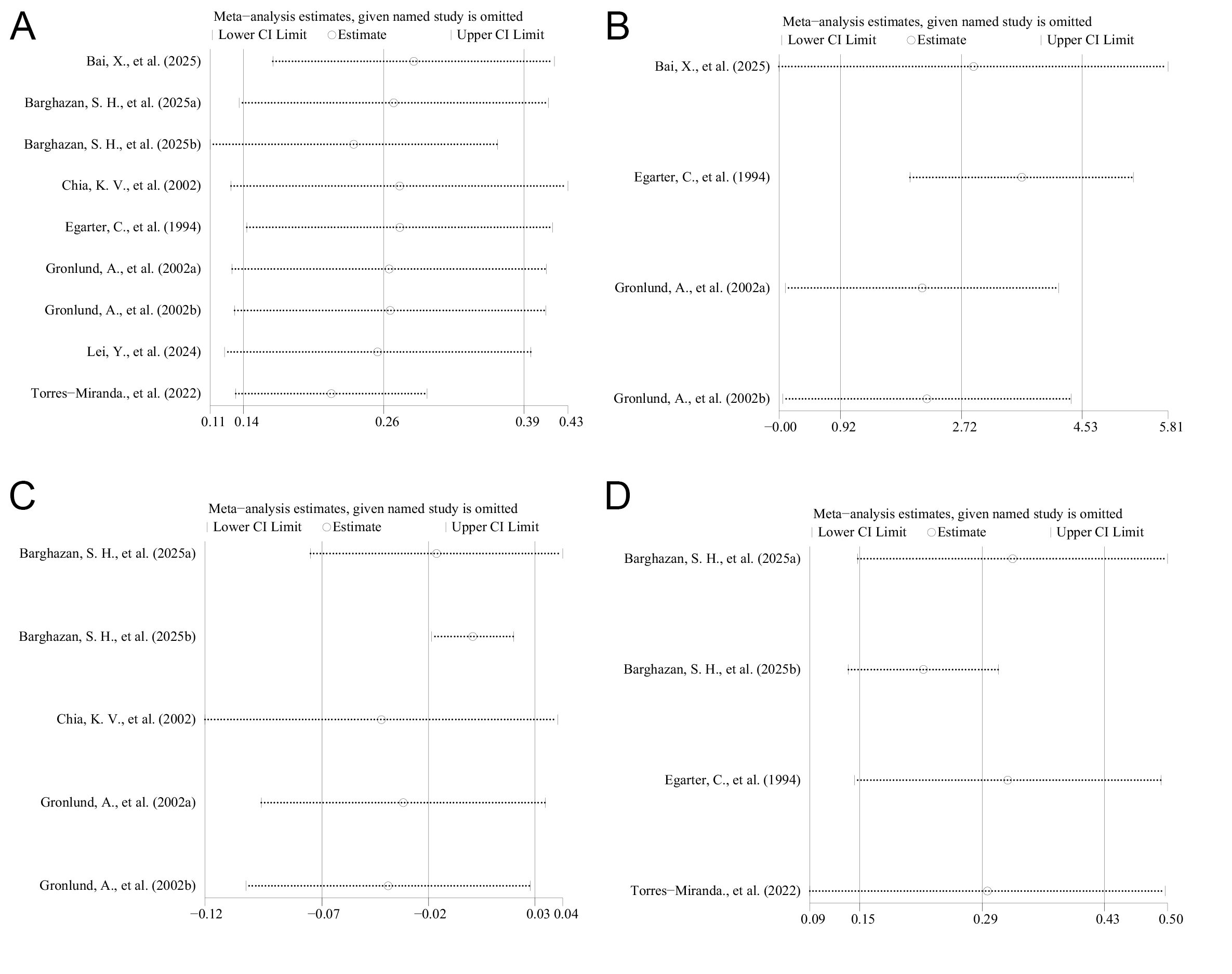

Supplement: Supplementary file 2 [file Image_2.TIF]

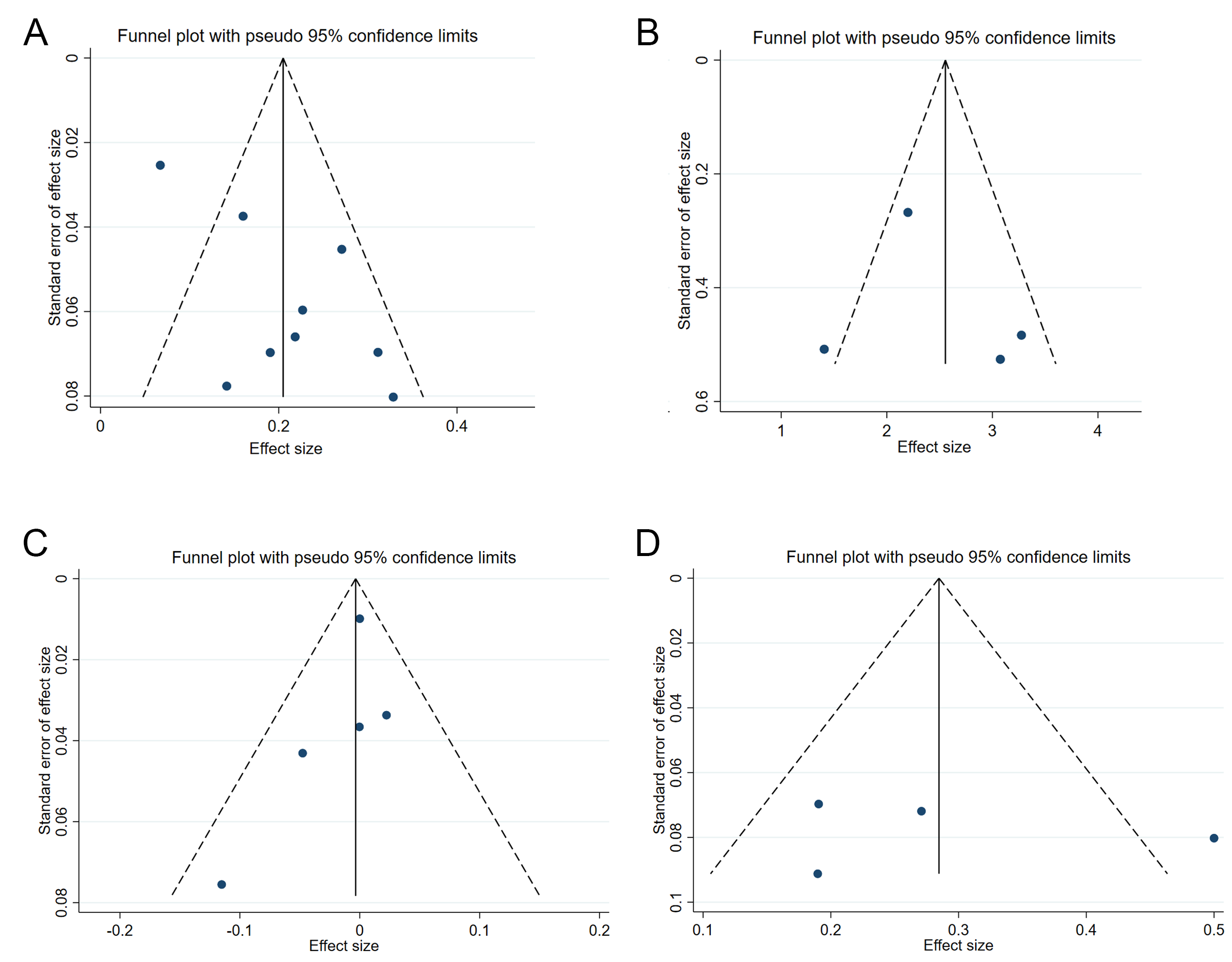

Supplement: Supplementary file 3 [file Image_3.TIF]
