# Supplementary material for: Comparative efficacy and safety of medical and surgical management for missed miscarriage: a systematic review and meta-analysis
Source: Front Med (Lausanne). 2026 Apr 15;13:1801007. doi: 10.3389/fmed.2026.1801007 (PMC13125087; doi:10.3389/fmed.2026.1801007)
Supplement: Supplementary file 4 [file Supplementary_file_1.docx]

Table S1. Search strategy of databases

| Databases | Search strategy | Published date | Results |
| --- | --- | --- | --- |
| PubMed | (((((missed miscarriage[Title/Abstract])) OR (missed abortion[Title/Abstract])) OR (delayed miscarriage[Title/Abstract])) AND ((((((((mifepristone[Title/Abstract])) OR (misoprostol[Title/Abstract])) OR (medical abortion[Title/Abstract])) OR (medical management [Title/Abstract])) OR (pharmacological treatment[Title/Abstract])) OR (drug therapy[Title/Abstract])) OR (medical treatment[Title/Abstract]))) AND ((((((((dilation and curettage[Title/Abstract])) OR (D&C[Title/Abstract])) OR (surgical evacuation[Title/Abstract])) OR (uterine curettage[Title/Abstract])) OR (vacuum aspiration[Title/Abstract])) OR (surgical management[Title/Abstract])) OR (surgical treatment[Title/Abstract])) | 1948-2026 | 61 |
| Web of science | missed miscarriage OR missed abortion OR delayed miscarriage (All Fields) AND mifepristone OR misoprostol OR medical abortion OR medical management OR pharmacological treatment OR drug therapy OR medical treatment (All Fields) AND dilation and curettage OR D&C OR surgical evacuation OR uterine curettage OR vacuum aspiration OR surgical management OR surgical treatment (All Fields) | 1900-2026 | 266 |
| EMBASE | ((missed miscarriage or missed abortion or delayed miscarriage) and (mifepristone or misoprostol or medical abortion or medical management or pharmacological treatment or drug therapy or medical treatment) and ((dilation and curettage) or D&C or surgical evacuation or uterine curettage or vacuum aspiration or surgical management or surgical treatment)).af. | 1974-2026 | 254 |
| ScienceDirect | (TITLE-ABS-KEY (missed miscarriage) OR TITLE-ABS-KEY (missed abortion)) AND (TITLE-ABS-KEY (medical management) OR TITLE-ABS-KEY (drug therapy) OR TITLE-ABS-KEY (medical treatment)) AND (TITLE-ABS-KEY (dilation and curettage) OR TITLE-ABS-KEY (surgical treatment)) | 1997-2026 | 189 |
