# Supplementary material for: Comparative efficacy and safety of medical and surgical management for missed miscarriage: a systematic review and meta-analysis
Source: Front Med (Lausanne). 2026 Apr 15;13:1801007. doi: 10.3389/fmed.2026.1801007 (PMC13125087; doi:10.3389/fmed.2026.1801007)
Supplement: Supplementary file 5 [file Supplementary_file_2.docx]

| Table S2. Quality assessment based on the Newcastle-Ottawa Scale (NOS) checklist. | | | | | | | | | | |
| --- | --- | --- | --- | --- | --- | --- | --- | --- | --- | --- |
| **Study** | **Selection** | | | |  | **Comparability** |  | **Outcome** | | **Total score** |
|  | Representativeness of the sample | Sample size | Non-respondent | Ascertainment of the exposure |  | Confounding factors are controlled |  | Assessment of outcomes | Statistical test |  |
| Bai, X., et al. 2025 | * | * | * | * |  | ** |  | * | * | 8 |
| Lei, Y., et al. 2024 | * | * | * | * |  | * |  | * | * | 7 |
| Torres-Miranda., et al. 2022 | * | * | * | * |  | * |  | * | * | 7 |
